# Inter-Model Variability of ChatGPT, Gemini and Claude in Treatment Recommendations for Unruptured Intracranial Aneurysms

**DOI:** 10.1007/s10143-026-04498-1

**Published:** 2026-09-16

**Authors:** Anish Narayan, Frederick Mariajoseph, Malik Farooq, Adrian Praeger, Ronil Chandra, Idrees Sher, Lee-Anne Slater, Calvin Gan, Andrew Gauden, Hamed Asadi, Justin Moore

**Affiliations:** 1 Faculty of Medicine, Monash University, Clayton, Australia; 2 Department of Neurosurgery, Monash Health, Clayton, Australia; 3 Department of Surgery, School of Clinical Sciences at Monash Health, Monash University, Melbourne, VIC Australia; 4 Monash Health Imaging, Monash Health, Melbourne, Australia; 5 Department of Radiology, Austin Health, Heidelberg, VIC 3084 Australia

**Keywords:** Unruptured intracranial aneurysms, Large language models, Inter-model variability, Artificial intelligence, Multidisciplinary team

## Abstract

Patients increasingly consult large language models (LLMs) before specialist review, yet whether frontier models agree with one another in nuanced clinical domains such as unruptured intracranial aneurysm (UIA) management remains uncharacterised. We therefore quantified inter-model variability across ChatGPT, Gemini and Claude, anchored against neurovascular multidisciplinary team (MDT) consensus and the Unruptured Intracranial Aneurysm Treatment Score (UIATS). Sixty-seven UIA cases referred to our neurovascular service (January–December 2025) were retrospectively analysed. De-identified clinical vignettes were submitted to Claude Opus 4.6, ChatGPT-5.4 and Gemini 3 Pro Thinking, each run five times. Within-model reproducibility was assessed using Fleiss’ κ; inter-model agreement via Cohen’s κ and McNemar’s test; and each LLM’s majority-vote anchored against MDT and UIATS using the same methods. Within-model reproducibility was almost perfect (Fleiss’ κ 0.837–0.860). Pairwise inter-model agreement was asymmetric: ChatGPT–Gemini behaved near-identically (Cohen’s κ = 0.850, 95% CI 0.71–0.97), whereas Claude diverged from both (κ = 0.688 and 0.667). Recommendations were non-unanimous in 13/67 cases (19.4%); Claude was the sole outlier in 8/13 (conservative in 7). Gemini was significantly more pro-treatment than Claude (McNemar *p* = 0.0117). Against MDT, Gemini and ChatGPT showed significant pro-treatment propensity (*p* = 0.0022, *p* = 0.0153). Claude was significantly more conservative than UIATS (*p* = 0.0162). Frontier LLMs are highly reproducible internally but diverge from one another asymmetrically. ChatGPT and Gemini behave near-identically while Claude diverges conservatively. Clinicians should anticipate AI-driven treatment expectations and future work should explore prompting, patient sentiment, and multicentre moderators.

**Supplementary Information:**

The online version contains supplementary material available at https://doi.org/10.1007/s10143-026-04498-1.

## Introduction

Unruptured intracranial aneurysms (UIAs) present one of the more nuanced decision-making challenges in neurovascular practice. Management options including pharmaceutical therapy, endovascular coiling, surgical clipping, or observation must be weighed against patient factors such as anxiety, pre-existing comorbidities, and aneurysm-specific characteristics, which together can render the identification of an optimal strategy particularly challenging [22]. Decisions are therefore carefully guided by skilled neurosurgeons and interventionalists, with advances in the field enabling increasingly personalised care tailored to the individual. Yet the context in which these decisions unfold has shifted. The advent and growing accessibility of large language models (LLMs) such as ChatGPT, Gemini, and Claude, along with health-specific tools like ChatGPT Health [19], has marked a revolution in how patients gather medical information, with many turning to AI to interpret complex material and form views on their diagnoses and treatment options before receiving specialist opinion [7, 23].

Existing literature has focused predominantly on independent human expert ratings of LLM outputs against clinical guidelines [13], or on the accuracy of older LLM models [20] given patient characteristics and input scores such as UIATS [10], PHASES [12] and ELAPSS [4]. However, these studies have largely overlooked a critical gap: they have not specifically analysed the inherent treatment propensities of the LLM models in comparison to expert consensus, nor the clinical and aneurysmal factors that may drive them. This is of direct relevance to neurovascular clinicians who must increasingly navigate and respond to AI-driven patient expectations.

We therefore conducted this study primarily to characterise inter-model variability in frontier LLM treatment recommendations for UIAs, quantifying both within-model reproducibility and pairwise between-model agreement across ChatGPT, Gemini, and Claude. Secondarily, we anchored each model’s treatment propensity against two independent clinical reference points including our neurovascular multidisciplinary team (MDT) consensus and UIATS to ascertain the treatment tendencies of each model and find correlating patient and aneurysmal factors.

## Materials and Methods

### Case selection

This study was approved by the institutional review board (RES-25-0000-885Q) and reported in accordance with the TRIPOD + LLM guidelines. All patient cases referred to our neurovascular service between January and December 2025 were reviewed. Included cases were adults (≥ 18 years) with at least one unruptured saccular intracranial aneurysm for whom the MDT decision (treat vs. observe) and, where applicable, the preferred treatment modality was recorded. Cases were excluded if the aneurysm was ruptured at presentation, if the patient was paediatric, or if clinical documentation was insufficient to populate the standardised prompt template.

### Data extraction and de-identification

Two reviewers (MF and AN) extracted clinical variables from the electronic medical record, MDT minutes, and radiology reports into a bespoke spreadsheet, with discrepancies resolved by consensus. Extracted variables included patient demographics, aneurysm characteristics, multiplicity, recurrence status, risk factors, functional status (modified Rankin Scale [mRS]), patient anxiety or treatment preference, prior imaging availability, interval since last follow-up, interval growth, and salient comorbidities. The reference-standard MDT outcome was recorded as a three-level categorical variable: NOT TREAT, TREAT (COIL), or TREAT (CLIP). Prior to any LLM interaction, all direct identifiers were removed and dates were converted to ages and intervals.

### UIATS scoring

Each case was independently scored using the UIATS [10]. We selected UIATS [10] because, unlike PHASES [12] and ELAPSS [4] which predict rupture risk and aneurysm growth respectively, it uniquely provides a specific management recommendation, allowing direct comparisons with both MDT decisions and LLM outputs. Scores were categorised as TREAT (net positive favouring intervention), NOT TREAT (net positive favouring observation), or UNCLEAR (equivocal). For cases with multiple aneurysms receiving discordant UIATS recommendations, MIXED was assigned.

### Large language models and prompting strategy

**Fig. 1:**
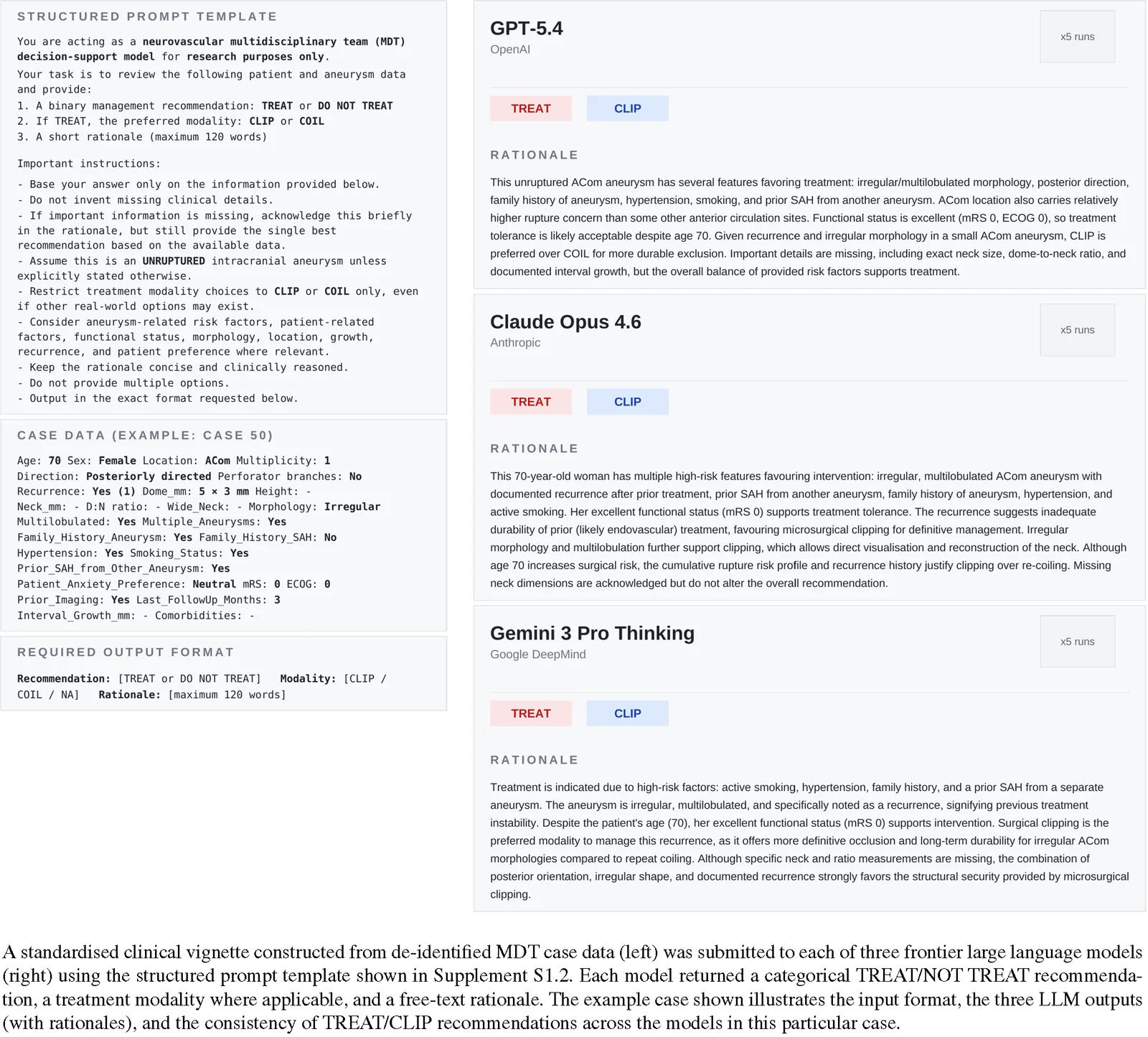
Prompting workflow for a representative case

Three frontier LLMs were evaluated: Claude Opus 4.6 (Anthropic), ChatGPT-5.4 (OpenAI), and Gemini 3 Pro Thinking (Google DeepMind), each accessed via its respective web interface. Extended-thinking mode was enabled for Claude (“Opus 4.6 Extended”) and Gemini; the standard ChatGPT-5.4 interface was used. No custom system instructions, fine-tuning, or retrieval-augmented generation was employed; web browsing and memory features were disabled for all models. Temperature, max token limits, and seed values were used as platform defaults. Each de-identified case was converted into a standardised clinical vignette and presented to each model using the structured prompt shown in Fig. 1. Critically, every model received byte-identical input vignettes, such that any between-model variation in output reflects model-intrinsic behaviour rather than input asymmetry. Detailed information regarding prompting, inference settings, output classifications and compute resources are present in Supplement S1–4.

### Outcomes

The primary outcome was pairwise inter-model agreement on the binary (TREAT vs. NOT TREAT) and three-level (NOT TREAT, TREAT (COIL), TREAT (CLIP)) treatment decision. Secondary outcomes included within-model reproducibility, agreement with MDT consensus and UIATS, and the direction and magnitude of any directional disagreement.

### Statistical analysis

Descriptive statistics were reported as counts and percentages with exact (Clopper–Pearson) 95% confidence intervals (CIs) for proportions.

- **Within-model reproducibility.** Within each LLM, the five per-case responses were treated as ratings from five raters across three nominal categories. Fleiss’ κ was computed; the proportion of cases with unanimous (5/5) agreement was also reported.
- **Inter-model agreement.** Pairwise Cohen’s κ was computed between each LLM pair (ChatGPT–Gemini, ChatGPT–Claude, Gemini–Claude) on majority-vote decisions, for both the binary and three-level outcomes, with bootstrap 95% CIs (5,000 replicates). The proportion of cases with unanimous three-model agreement was reported. McNemar’s test (exact when discordant pairs < 25) assessed directional asymmetry between each pair.
- **Agreement with MDT and UIATS.** Each LLM’s majority-vote was compared with the MDT decision using Cohen’s κ (three-level and binary) and with definitive UIATS classifications using binary κ. κ values were interpreted using Landis and Koch (< 0.00 poor; 0.00–0.20 slight; 0.21–0.40 fair; 0.41–0.60 moderate; 0.61–0.80 substantial; 0.81–1.00 almost perfect).
- **Directional treatment preference.** McNemar’s test was applied to the 2 × 2 table of discordant pairs against MDT and UIATS to test whether disagreements were directionally symmetric.
- **Subgroup analysis.** Cases where the LLMs universally disagreed with MDT were reviewed in depth to identify information selectively available to MDTs.

All analyses were performed in R; statistical significance set at α = 0.05 (two-tailed).

## Results

A total of 67 cases harbouring 99 aneurysms were identified. The MDT recommended observation in 49 (73.1%) and intervention in 18 (26.9%; coiling 10, clipping 8). UIATS yielded a definitive TREAT in 21 (31.3%), definitive NOT TREAT in 19 (28.4%), UNCLEAR in 18 (26.9%), and MIXED in 9 (13.4%). Excluding UNCLEAR and MIXED, 40 cases formed the LLM-versus-UIATS analytic subset. Baseline characteristics are presented in Table 1.

**Table 1:** Baseline demographic, clinical, and aneurysm characteristics

Characteristic	Value
Patient demographics (*n* = 67)	
Age, years, median (IQR)	61 (53–70.5)
Sex	
Female	52 (77.6%)
Male	15 (22.4%)
Detection mode	
Incidental	64 (95.5%)
Surveillance	2 (3.0%)
Screening	1 (1.5%)
Risk factors (*n* = 67)	
Hypertension	36 (53.7%)
Smoking status	
Current	19 (28.4%)
Former	14 (20.9%)
Never/no	34 (50.7%)
Family history of aneurysm	10 (14.9%)
Family history of SAH	6 (9.0%)
Prior SAH from another aneurysm	14 (20.9%)
Functional status (*n* = 67)	
mRS, median (IQR)	0 (0–1)
0	47 (70.1%)
1	13 (19.4%)
2	3 (4.5%)
≥ 3	2 (3.0%)
Not documented	2 (3.0%)
Comorbidities documented	11 (16.4%)
Patient preference (*n* = 67)	
Neutral or not stated	63 (94.0%)
Anxiety regarding aneurysm	3 (4.5%)
Strong observation preference	1 (1.5%)
Aneurysm characteristics (*n* = 99)	
Multiple aneurysms per patient	21/67 patients (31.3%)
Recurrent aneurysm	11 (11.1%)
Irregular morphology	10 (10.1%)
Multilobulated	5 (5.1%)
Perforator branches from neck/dome	3 (3.0%)
Location	
Internal carotid artery	34 (34.3%)
Middle cerebral artery	21 (21.2%)
Anterior communicating artery	12 (12.1%)
Posterior communicating artery	11 (11.1%)
Basilar artery	7 (7.1%)
Anterior cerebral artery/pericallosal	7 (7.1%)
Posterior cerebral artery	4 (4.0%)
Superior cerebellar artery	3 (3.0%)
Circulation	
Anterior	74 (74.7%)
Posterior	25 (25.3%)
MDT consensus decision (*n* = 67)	
Observation (NOT TREAT)	49 (73.1%)
Intervention (TREAT)	18 (26.9%)
Endovascular coiling	10 (55.6% of treated)
Microsurgical clipping	8 (44.4% of treated)
UIATS classification (*n* = 67)	
TREAT	21 (31.3%)
NOT TREAT	19 (28.4%)
UNCLEAR	18 (26.9%)
MIXED	9 (13.4%)

Data are presented as n (%) unless otherwise specified. Patient-level variables (demographics, risk factors, functional status, preference) use a denominator of 67 patients. Aneurysm-level variables (morphology, location) use a denominator of 99 aneurysms. Modality percentages are reported as a proportion of the 18 treated cases. UIATS categories: TREAT, all aneurysms in the case favoured intervention; NOT TREAT, all aneurysms favoured observation; UNCLEAR, all aneurysms were equivocal; MIXED, the case contained multiple aneurysms with discordant UIATS recommendations across individual lesions

*IQR* interquartile range, *mRS* modified Rankin Scale, *SAH* subarachnoid haemorrhage

All three LLMs demonstrated almost perfect within-model reproducibility (Fleiss’ κ 0.837–0.860; ChatGPT highest at κ = 0.860). Pairwise between-model agreement on the binary decision was, however, asymmetric: ChatGPT and Gemini showed almost perfect concordance (Cohen’s κ = 0.850, 95% CI 0.71–0.97; 62/67 = 92.5%), whereas Claude diverged substantially from both ChatGPT (κ = 0.688, 95% CI 0.50–0.85; 57/67 = 85.1%) and Gemini (κ = 0.667, 95% CI 0.48–0.84; 56/67 = 83.6%). Unanimous three-model agreement on the binary decision was observed in 54/67 cases (80.6%, 95% CI 69.1–89.2), with at least one model diverging in 13/67 (19.4%, 95% CI 10.8–30.9). Claude was the sole outlier in 8 of the 13 non-unanimous cases, recommending observation against a pro-treatment ChatGPT–Gemini consensus in 7 of these 8. Pairwise McNemar’s tests confirmed this asymmetry with Gemini significantly more pro-treatment than Claude (*p* = 0.0117) and ChatGPT trending pro-treatment relative to Claude (*p* = 0.109).

Anchored against MDT consensus and UIATS, three-way agreement with MDT was comparable across the three LLMs, with the highest agreement observed for Gemini (Cohen’s κ = 0.380). Binary agreement with MDT ranged from 71.6% (Claude) to 74.6% (ChatGPT), and against UIATS from 72.5% (Claude) to 75.0% (ChatGPT and Gemini), with Cohen’s κ 0.461–0.504 (Table 2). UIATS and MDT consensus themselves significantly diverged on the 40 definitive cases (McNemar’s χ² = 4.571, *p* = 0.033), with UIATS recommending treatment in 21/40 (52.5%) versus 13/40 (32.5%) by MDT, demonstrating that the UIATS recommendations were significantly pro-treatment relative to MDT consensus. These are detailed in Tables 2 and 3.

**Table 2:** Head-to-head concordance comparison of Gemini, Claude, and ChatGPT outputs against MDT and UIATS baselines

Domain	Metric	Gemini	Claude	ChatGPT
***Internal reproducibility***	N cases	67	67	67
	Unanimous 5/5	55/67; 82.1%, [70.8–90.4]	56/67; 83.6%, [72.5–91.5]	54/67; 80.6% [69.1–89.2]
	Fleiss’ κ	0.857	0.837	**0.860**
***LLM vs. MDT***	Exact 3-level agreement	44/67; 65.7% [53.1–76.8]	43/67; 64.2% [51.5–75.5]	43/67; 64.2% [51.5–75.5]
	Cohen’s κ (3-level)	**0.380**	0.242	0.322
	Binary agreement (Treat/Not Treat)	49/67; 73.1% [60.9–83.2]	48/67; 71.6% [59.3–82.0]	50/67; 74.6% [62.5–84.5]
	Cohen’s κ (binary)	0.451	0.337	**0.459**
***LLM vs. UIATS***	Definitive UIATS cases	40	40	40
	Binary agreement	30/40; 75.0% [58.8–87.3]	29/40; 72.5% [56.1–85.4]	30/40; 75.0% [58.8–87.3]
	Cohen’s κ (binary)	0.501	0.461	**0.504**

Values in bold denote the highest-performing model for that metric (agreement, kappa). Significant p-values (< 0.05) are also bolded. Confidence intervals are exact Clopper–Pearson 95% CIs

*CI* confidence interval*, κ*kappa

**Table 3:** Pairwise inter-model agreement between Gemini, Claude, and ChatGPT

Domain	Model pair	Binary agreement (κ [95% CI])	3-level agreement (κ)
*Pairwise inter-model agreement*	ChatGPT–Gemini	62/67; 92.5% (κ = 0.850 [0.71–0.97])	57/67; 85.1% (κ = 0.751)
	ChatGPT–Claude	57/67; 85.1% (κ = 0.688 [0.50–0.85])	53/67; 79.1% (κ = 0.624)
	Gemini–Claude	56/67; 83.6% (κ = 0.667 [0.48–0.84])	52/67; 77.6% (κ = 0.611)
*Inter-model directional asymmetry (McNemar)*	ChatGPT vs. Gemini	*p* = 0.375 (symmetric)	—
	ChatGPT vs. Claude	*p* = 0.109 (trend; ChatGPT more pro-treatment)	—
	Gemini vs. Claude	*p* = 0.0117 (Gemini more pro-treatment)	—
*Three-way concordance*	All three models	Unanimous binary: 54/67; 80.6% [69.1–89.2]	Unanimous 3-level: 48/67; 71.6%

Values in bold denote the highest-performing model for each metric or the statistically significant inter-model comparison. Confidence intervals are exact Clopper–Pearson 95% CIs for proportions and bootstrap 95% CIs (5,000 replicates) for pairwise κ. McNemar’s test uses the exact binomial when discordant pairs < 25

*CI* confidence interval*; κ*kappa

**Fig. 2:**
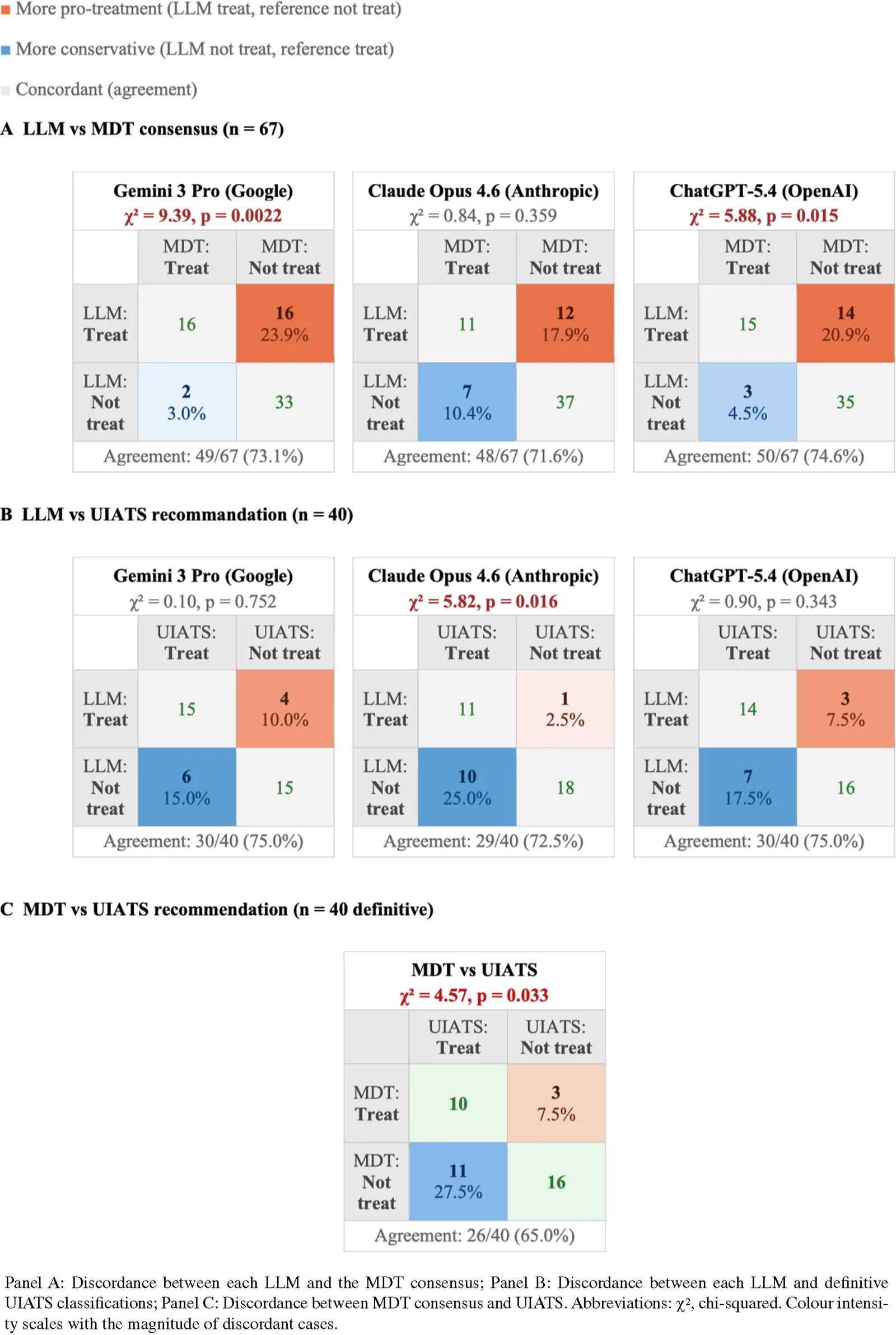
McNemar 2 × 2 contingency tables depicting directional treatment disagreement. Panel **A**: Discordance between each LLM and the MDT consensus; Panel **B**: Discordance between each LLM and definitive UIATS classifications; Panel **C**: Discordance between MDT consensus and UIATS. Abbreviations: χ², chi-squared. Colour intensity scales with the magnitude of discordant cases

Each LLM’s directional propensity against these anchors recapitulated the inter-model pattern. Gemini and ChatGPT recommended treatment significantly more often than MDT (McNemar’s *p* = 0.0022 and *p* = 0.0153, respectively), while Claude showed no significant directional disagreement against MDT but was significantly more conservative than UIATS (*p* = 0.0162). Treatment preferences are visualised in Fig. 2.

In the UNCLEAR UIATS subgroup, the MDT overwhelmingly favoured observation (15/18; 83.3%), and all three LLMs largely mirrored this conservatism (72.2–77.8%), indicating the pro-treatment propensity of Gemini and ChatGPT is driven by definitive cases rather than equivocal ones. In the 10 universally discordant pro-treatment cases (all three LLMs treat, MDT observes), a high burden of treatment-favouring risk factors was observed including 50.0% with prior SAH, 40.0% recurrent aneurysms, and 50.0% multiple aneurysms; none carried a definitive UIATS of NOT TREAT.

However, review of the corresponding MDT records revealed that in every case the MDT had access to clinically meaningful information not captured in the structured vignette including patient comorbidity and procedural risk (cases 3, 6, 7, 9), prior MDT meetings and treatment trajectories (cases 1, 2, 5, 8, 10), morphological nuance (case 4), and adherence concerns (cases 4, 8). These are detailed in Table 4.

**Table 4:** Detailed clinical and radiological profiles of the ten universally discordant pro-treatment cases

Case	Age/Sex	Aneurysm(s) — location & size	Treatment-favouring features visible to LLM	Likely factors moderating MDT towards observation	Comorbidities documented	UIATS	mRS
1	61/F	L ICA terminus aneurysm (recurrent) and small L PCA aneurysm; surveillance imaging stable	Multiple aneurysms; recurrence (×2 retreatments in 2004 and 2009); prior SAH from ruptured L carotid terminus	*Long-term surveillance patient with stable lesions on most recent MRI (19/1/25); both aneurysms small; multiple prior interventions favouring continued imaging follow-up*	*None documented*	TREAT	0
2	55/F	Multiple: clipped R supraclinoid ICA (3.5 mm neck remnant), coiled basilar tip (3.5 mm neck remnant, retreated 2023), bilobed R ophthalmic ICA (4.2 × 7.2 × 5.9 mm, partially intradural), tiny R anterior choroidal (0.5 × 0.8 mm)	Prior SAH; multiple aneurysms; current smoker; recurrence (×2); multilobulated bilobed morphology; ophthalmic aneurysm 7 mm	*Most aneurysms already treated with stable neck remnants; only the ophthalmic ICA aneurysm remains untreated and unchanged on imaging; ongoing satisfactory occlusion of treated lesions argues against further intervention*	*None documented*	TREAT (Basilar artery)/UNCLEAR (R ICA)	0
3	71/F	L superior hypophyseal ICA aneurysm with documented growth from 2.2 mm to ~ 4 mm over 2 years	Documented interval growth; multiple aneurysms; older patient with growth on serial imaging	*MDT noted only “slight growth over 2 years (2.2 mm → 4 mm)” in a 71-year-old with cerebellar atrophy; small absolute size and indolent growth in an elderly patient with neurological vulnerability*	*Cerebellar atrophy noted in MDT discussion (not in vignette)*	TREAT	0
4	58/F	L ICA aneurysm 8 × 6.5 mm at neck, arising below the ophthalmic artery, directed inferiorly — morphologically extradural	Size > 5 mm; current smoker (10/day, very reluctant to stop); reported headaches	*Previous MDT decision: “Extradural aneurysm — small. Explain CN symptoms. Plan to monitor with another MRI in 12 months*,* if stable can probably relax to 2-yearly.” Extradural location carries lower rupture risk; patient DNA ×2 (20/5 and 10/6); night-shift worker*	*None documented*	TREAT	0
5	52/M	PCOM aneurysm — 2006 embolisation, 2022 recurrence treated with Pipeline Vantage flow diverter, residual remnant on Dec 2023 imaging	Recurrence; prior treatment history; PCOM location; hypertension; ex-smoker	*MRI 7/4/25 showed “near-complete occlusion” of the aneurysm post flow-diverter treatment; MDT decision was explicitly “continue surveillance” as the device is doing its job*	*None documented*	UNCLEAR	0
6	57/F	WFNS 1 SAH from ruptured R pericallosal aneurysm treated with Artisse 12/8/25; residual untreated 3 mm L MCA and 3 mm ophthalmic segment aneurysms	Prior SAH; family history of both aneurysm and SAH; multiple aneurysms	*Both untreated lesions only 3 mm; patient is non-smoker with normal BP; MDT explicitly raised “suitability for surveillance” as the question; small size in a low-risk patient favours imaging follow-up*	*None documented*	TREAT (ICA)/UNCLEAR (MCA)	0
7	52/F	Recurrent ACOM aneurysm at neck (3.0 × 3.2 mm, slowly enlarging from 2.7 × 2.7 mm); stable R SCA 2 mm; extradural R ICA 2.8 × 1.9 mm	Prior SAH (WFNS 1, 2022); multiple aneurysms (3); recurrence; hypertension; current smoker	*Patient has progressive degenerative neurological/dementia condition under Neurology and Neuropsychiatry; described as verbally aggressive and emotionally labile in clinic — capacity and procedural risk concerns. MDT decision: “MRI surveillance”*	*Dementia/progressive degenerative nervous system disease (not in vignette)*	TREAT	2
8	66/M	R PCOM 3 mm (stable), L PCOM 2.5 mm (stable), R pericallosal 2 mm (stable), all unchanged from 2021	Multiple aneurysms (3); irregular morphology of R PCOM; family history of both aneurysm and SAH	*All three aneurysms small (≤ 3 mm) and stable for 4 years; previous MDT decision (29/4/25): “if attends*,* consider MRI/MRA*,* if DNA then discharge”. Patient repeatedly cancels appointments and scans; engagement and adherence concerns*	*None documented*	TREAT (PCOM)/UNCLEAR (pericallosal)	0
9	53/M	Coiled R terminal ICA aneurysm (posterolateral component, ruptured 12/2023); recurrence retreated 11/12/24 with post-procedural left-sided face/arm/leg weakness; plate-like residual at base of coiled R PCA aneurysm (~ 9 × 7 × 1.5 mm)	Prior SAH; recurrence; hypertension; large residual	*Patient suffered a peri-procedural stroke from the most recent intervention with residual deficit; severe atherosclerosis and ongoing cardiomyopathy substantially increase procedural risk; further intervention would carry high morbidity*	*Severe atherosclerosis*,* ongoing cardiomyopathy (not in vignette)*	TREAT (PCA)/UNCLEAR (ICA)	2
10	70/F	L MCA aneurysm 5.5 × 4.9 × 4.1 mm, stable on MRI 15/4/25, “slightly larger” compared with June 2019 baseline	Documented (very slow) interval growth over 6 years; MCA bifurcation location	*Older patient (70) with minimal growth over 6 years (essentially stable); MDT decision: “continue surveillance”*	*None documented*	UNCLEAR	0

## Discussion

### Findings

To our knowledge, this is the first study to systematically characterise inter-model variability in frontier LLM treatment recommendations for UIAs. Despite almost perfect within-model reproducibility, between-model agreement was asymmetric: ChatGPT and Gemini behaved near-identically, whereas Claude diverged substantially from both, emerging as the sole conservative outlier in 7 of 8 non-unanimous cases. Anchored against MDT and UIATS, these divergences resolved into reproducible behavioural archetypes: ChatGPT and Gemini exhibited significant pro-treatment propensities relative to MDT, whereas Claude was significantly more conservative than UIATS. Pairwise McNemar testing directly confirmed Gemini’s significant pro-treatment propensity relative to Claude. These differences are arguably more clinically consequential than any single model’s absolute agreement with expert consensus, because they imply that a patient’s AI-derived treatment expectation is, in part, an artefact of which model they happened to consult before specialist review.

In comparison to the LLMs, our MDT was significantly more conservative, reflecting decades of UIA landmark trials including UIATS [10], PHASES [12], ELAPSS [4], ISUIA [14], and UCAS [26], collectively integrated during MDT deliberation. Central to these trials is the consistent finding that annual rupture risk is approximately 0.5–1% [4, 10, 12, 14, 26], a figure low enough that for many patients, particularly those with small, anterior circulation aneurysms without high-risk morphology, procedural risk may equal or exceed observation risk. This evidence-grounded conservatism is the principal reason we considered MDT consensus an appropriate clinical anchor. Furthermore, whilst our MDT data appeared more conservative than expected, this was not considered a significant biasing limitation, considering that the typical range of conservative treatment for UIAs can range from 38% to 70% depending on demographic and location [11, 15, 27].

Our subgroup analysis further showcased that MDT divergence from LLM outputs is grounded in clinical judgement and contextual factors. In universally discordant cases, factors pushing the MDT towards conservatism included patient comorbidities (cases 3, 7, 9), prior MDT decisions and treatment trajectories (cases 1, 2, 5, 8, 10), morphological nuance such as Case 4’s extradural carotid lesion conferring substantially lower rupture risk, and adherence concerns (cases 4, 8 with repeated appointment cancellations). Hence, part of the discordance reflected historical and contextual patient information unbeknownst to the LLMs, which received inputs as if cases were first presentations.

The inter-model differences in treatment propensity can be reasoned through interacting factors including each model’s algorithmic design, training-data asymmetries, and reliance on formal risk-stratification anchors. LLMs have an inherent programmatic optimisation for immediate user satisfaction [5, 16], a trait often referred to as “sycophancy” [9], which in clinical context can manifest as a preference for definitive, actionable recommendations over ambiguous alternatives such as continued observation. The pro-treatment stance of ChatGPT and Gemini is consistent with a “Default Value Orientation” towards active intervention [6], and the GPT family has demonstrated similar behaviour across clinical domains [13, 24]. Claude’s significant conservative position likely stems from its constitutional framework [3] which mandates Claude to choose the response that is “least threatening or pro-treatment.” Methodologically, our vignettes allowed the LLMs to anchor in formal scoring systems, and given UIATS demonstrated a higher propensity for treatment than MDT in our cohort, it is plausible that Gemini and ChatGPT mirrored that preference — strengthened by our finding that in every universally discordant pro-treatment case, UIATS produced a recommendation compatible with intervention. A further explanation lies in training-corpora asymmetries: the published neurovascular literature is disproportionately weighted towards interventional outcomes and procedural case series [17], an imbalance that could contribute to the pro-treatment propensity of ChatGPT and Gemini. Together these hypotheses reframe inter-model variability not as a flaw in any single model’s reasoning, but as a predictable consequence of distinct design, data, and alignment choices operating on a common clinical input.

### Patient implications

The choice to treat versus monitor is no benign undertaking. Endovascular treatment carries a procedural complication risk of 4.96% with a case-fatality rate of 0.30% [2], whilst microsurgical approaches carry a 13.9% risk of poor neurological outcomes and 24.1% all-cause adverse events [25]. Conversely, undertreatment driven by conservative LLMs may leave patients vulnerable to SAH. As ChatGPT remains the most-used LLM at the time of this paper, it’s pro-treatment preference may exacerbate overtreatment risks, particularly as the traditional dualistic doctor-patient relationship rapidly evolves into a complex triad of doctor, patient, and AI [8]. This is especially problematic given AI’s sycophantic nature and confident presentation, which can sway patients towards an option they are already leaning towards, particularly if the patient was already leaning towards that option. In blinded evaluations where authorship is concealed, patients consistently prefer AI-generated responses, rating them as more thorough, trustworthy, and empathetic than those written by human physicians [18]. Hence, it is possible that AI could sway departments towards overtreatment secondary to patient (and AI) pressure as we have already seen in antimicrobial prescription where patient insistence for antibiotics leads to overprescription [1].

Ultimately, capitulating to a patient’s AI-driven insistence could force clinicians into dilemmas between upholding evidence-based medicine, optimising resource utilisation and the pressure of patient autonomy. It may become difficult for clinicians to grapple with a patient’s AI-assisted predilections which could negatively harm them and convince them of otherwise without disrupting the therapeutic relationship.

### Limitations

The integration of LLMs into neurosurgery and interventional neuroradiology remains in its nascent stages, and the findings derived from our tertiary centre cohort must be contextualised within significant structural limitations. Firstly, our study evaluated a specific snapshot of three frontier models (Claude Opus 4.6, ChatGPT-5.4, Gemini 3 Pro) in 2026 and given the rapid, iterative nature of AI development, the specific personality traits and preferences of these models are subject to continuous algorithmic drift.

Furthermore, our retrospective design also precluded reliable capture of patient anxiety and emotional context common in cases of untreated UIAs [21]. This is further relevant given that these emotional factors can affect how patients interact with LLMs, with anxious patients potentially engaging with LLMs through leading questions. In these cases, LLM sycophancy may subtly optimise to provide a pleasing response (reinforcing what the patient wants to hear) rather than challenge such sentiments, entrenching potentially malignant treatment preference. The assessment of confirmation bias remains an important avenue for future work. Similarly, other patient variables that would have been readily available to the MDT during deliberation were not reliably extractable from the electronic record and were therefore omitted from the structured vignettes provided to the LLMs. These included comorbidity burden, anticoagulant or antiplatelet therapy, frailty, prior MDT decisions on the same patient, and patterns of treatment adherence which, as the case-level review of the ten universally discordant pro-treatment cases demonstrated, frequently underpinned the MDT’s decision to continue observation.

Finally, our rigid prompting structure, which patients would rarely present their cases as, and the binary treat/observe framing imposed on the LLMs inevitably oversimplifies a clinical reality in which equipoise is common and nuanced. MDT deliberation frequently ends not in a categorical recommendation, but in a shared decision that holds multiple possibilities open. In addition, whilst we compared decision-making frameworks, we didn’t assess outcome validation such as treatment complications which are also essential to consider.

### Future directions

Further research should prioritise integrating more in-depth comorbidity factors into LLM inputs such as providing Charlson Comorbidity Index scores and adapting LLM inputs with personality nuances to more accurately determine the LLM’s true capacity at determining an ideal evidence-based strategy. This could involve grading the patient’s level of anxiety regarding either treatment or observation and then manipulating the input response to reflect these sentiments such as by mentioning the preferred treatment option within the input. Further qualifiers in the input response such as “be evidence-based” or more personal nuances such as “I want to be able to continue running,” could be trialled to develop a more complete and comprehensive picture as to how LLM’s respond to patients. In addition, using multicentre datasets would also assist in creating a more holistic comparison that doesn’t accentuate treatment tendences existing within a single centre’s MDT.

## Conclusion

In this study, we have demonstrated that frontier LLMs, despite almost perfect within-model reproducibility, are not behaviourally interchangeable in their treatment recommendations for unruptured intracranial aneurysms. However, we also uncover that this between-model variability is asymmetric and directionally non-random. ChatGPT and Gemini behave near-identically and trend pro-treatment, whereas Claude diverges substantially from both and trends conservative — archetypes large enough that approximately one in five patients would receive a non-unanimous recommendation across the three models. Our data provide a clear rationale for clinicians to anticipate AI-driven treatment expectations as model-specific rather than monolithic, and for future work to interrogate how prompting design, patient sentiment, and multicentre case-mix modulate these archetypes.

## Supplementary Information

Below is the link to the electronic supplementary material.

Supplementary Material 1 (DOCX 21.3 KB)

Supplementary Material 2 (DOCX 28.7 KB)

## Data Availability

No datasets were generated or analysed during the current study.

## Code Availability

R scripts used for statistical analysis are available from the corresponding author upon reasonable request.

## Declarations

### Registration

This study was prospectively registered on the Open Science Framework (https://doi.org/10.17605/OSF.IO/358MQ).
