# Supplementary material for: Inter-Model Variability of ChatGPT, Gemini and Claude in Treatment Recommendations for Unruptured Intracranial Aneurysms
Source: Neurosurg Rev. 2026 Sep 16;49(1):569. doi: 10.1007/s10143-026-04498-1 (PMC13582248; doi:10.1007/s10143-026-04498-1)
Supplement: Supplementary file 1 [file 10143_2026_4498_MOESM1_ESM.docx]

# **Supplementary Methods: LLM Prompting, Annotation, and Inference Configuration**

## **S1. Prompt Design, Development, and Selection**

### **S1.1 Prompt development process**

The standardised clinical vignette prompt was developed de novo by two investigators (AN and MF) through an iterative process. An initial draft prompt was constructed based on the clinical variables routinely documented in neurovascular MDT case notes and refined through pilot testing on non-study cases selected from outside the January–December 2025 study period. These pilot cases were not included in the final 67-case analytic cohort. During pilot testing, the prompt was revised across three iterations to optimise: (1) the completeness of clinical variable capture, (2) the clarity and enforceability of output format constraints, and (3) the consistency of LLM responses across repeated runs. No alternative prompt architectures (e.g., chain-of-thought, few-shot, or role-play variants) were formally tested; the final prompt employed a zero-shot, structured-input approach with explicit output format constraints.

### **S1.2 Final prompt template**

The complete prompt template submitted to each LLM is reproduced verbatim below. For each of the 67 study cases, the bracketed placeholders (e.g., [AGE], [SEX], [LOCATION]) were populated with the corresponding de-identified patient and aneurysm data extracted from the MDT case notes. No additional system-level instructions, preambles, or follow-up messages were appended. Each prompt was submitted as a single-turn, standalone query in a new conversation session.

You are acting as a neurovascular multidisciplinary team (MDT) decision-support model for research purposes only.

Your task is to review the following patient and aneurysm data and provide:

1. A binary management recommendation: TREAT or DO NOT TREAT

2. If TREAT, the preferred modality: CLIP or COIL

3. A short rationale (maximum 120 words)

Important instructions:

- Base your answer only on the information provided below.

- Do not invent missing clinical details.

- If important information is missing, acknowledge this briefly in the rationale, but still provide the single best recommendation based on the available data.

- Assume this is an UNRUPTURED intracranial aneurysm unless explicitly stated otherwise.

- Restrict treatment modality choices to CLIP or COIL only, even if other real-world options may exist.

- Consider aneurysm-related risk factors, patient-related factors, functional status, morphology, location, growth, recurrence, and patient preference where relevant.

- Keep the rationale concise and clinically reasoned.

- Do not provide multiple options.

- Output in the exact format requested below.

-----------------------------------

CASE DATA

-----------------------------------

Age: [AGE]

Sex: [SEX]

Location: [LOCATION]

Location_Clean: [LOCATION_CLEAN]

Multiplicity: [MULTIPLICITY]

Direction_of_Aneurysm: [DIRECTION_OF_ANEURYSM]

Perforator_Branches_from_Neck_or_Dome: [PERFORATOR_BRANCHES]

Recurrence: [RECURRENCE]

Dome_mm: [DOME_MM]

Height_mm: [HEIGHT_MM]

Neck_mm: [NECK_MM]

Dome_to_Neck_Ratio: [DOME_TO_NECK_RATIO]

Wide_Neck: [WIDE_NECK]

Morphology: [MORPHOLOGY]

Multilobulated: [MULTILOBULATED]

Multiple_Aneurysms: [MULTIPLE_ANEURYSMS]

Family_History_Aneurysm: [FAMILY_HISTORY_ANEURYSM]

Family_History_SAH: [FAMILY_HISTORY_SAH]

Hypertension: [HYPERTENSION]

Smoking_Status: [SMOKING_STATUS]

Prior_SAH_from_Other_Aneurysm: [PRIOR_SAH_OTHER_ANEURYSM]

Patient_Anxiety_Preference: [PATIENT_ANXIETY_PREFERENCE]

mRS: [MRS]

ECOG: [ECOG]

Prior_Imaging: [PRIOR_IMAGING]

Last_FollowUp_Months: [LAST_FOLLOWUP_MONTHS]

Interval_Growth_mm: [INTERVAL_GROWTH_MM]

Comorbidities: [COMORBIDITIES]

-----------------------------------

REQUIRED OUTPUT FORMAT

-----------------------------------

Recommendation: [TREAT or DO NOT TREAT]

Modality: [CLIP / COIL / NA]

Rationale: [maximum 120 words]

*The structured prompt template above is also visualised in Fig. 1 of the main manuscript.*

### **S1.3 Prompt input variables**

The prompt template contained 27 structured clinical variables spanning five domains, as listed below. All variables were extracted from the electronic medical record, MDT minutes, and radiology reports by two reviewers (MF and AN). Where a variable was not documented in the clinical record, the field was populated with “Not documented” or “Not available” rather than left blank, to ensure the LLM did not infer missing data.

| **Domain** | **Variables** | **Data type / values** |
| --- | --- | --- |
| Demographics | Age, Sex | Continuous (years); Categorical (M/F) |
| Aneurysm morphology | Location, Location_Clean, Direction_of_Aneurysm, Perforator_Branches, Dome_mm, Height_mm, Neck_mm, Dome_to_Neck_Ratio, Wide_Neck, Morphology, Multilobulated | Categorical and continuous as appropriate |
| Multiplicity and recurrence | Multiplicity, Recurrence, Multiple_Aneurysms | Categorical (Yes/No) and free text |
| Patient risk factors | Family_History_Aneurysm, Family_History_SAH, Hypertension, Smoking_Status, Prior_SAH_from_Other_Aneurysm, mRS, Comorbidities | Categorical and continuous as appropriate |
| Clinical context | Patient_Anxiety_Preference, Prior_Imaging, Last_FollowUp_Months, Interval_Growth_mm | Categorical and continuous as appropriate |

## **S2. LLM Inference Configuration**

### **S2.1 Models and access method**

Three frontier LLMs were evaluated: Claude Opus 4.6 (Anthropic), ChatGPT-5.4 (OpenAI), and Gemini 3 Pro Thinking (Google DeepMind). All models were accessed via their respective consumer web interfaces (claude.ai, chatgpt.com, and gemini.google.com). No API access was used. All prompts were submitted in March 2026.

### **S2.2 Inference settings**

All models were accessed using platform default settings. Temperature, maximum token limits, seed values, and other inference hyperparameters could not be user-configured through the web interfaces. The documented API default temperature for all three models is 1.0; however, the web interface defaults are managed internally by each provider and may differ from API defaults. For Claude, the extended-thinking mode was enabled (“Opus 4.6 Extended”); for this mode, the temperature parameter is overridden by the thinking framework and does not apply. For Gemini, the thinking mode was enabled; Google recommends keeping temperature at its default value of 1.0 for Gemini 3 models, as reducing temperature below 1.0 may cause looping or degraded performance. For ChatGPT, the standard ChatGPT-5.4 interface was used; temperature is fixed and not user-adjustable in the standard web interface.

| **Parameter** | **Claude Opus 4.6** | **ChatGPT-5.4** | **Gemini 3 Pro Thinking** |
| --- | --- | --- | --- |
| Access method | Web interface (claude.ai) | Web interface (chatgpt.com) | Web interface (gemini.google.com) |
| Temperature | Default (1.0 API); overridden by extended thinking | Default (1.0 API); fixed in web UI | Default (1.0 API); recommended for Gemini 3 |
| Max tokens | Platform default (not user-configurable) | Platform default (not user-configurable) | Platform default (not user-configurable) |
| Seed | Not available in web interface | Not available in web interface | Not available in web interface |
| Top-p / Top-k | Not user-configurable | Not user-configurable | Not user-configurable |
| Extended thinking | Enabled (“Opus 4.6 Extended”) | N/A | Enabled (thinking mode) |
| System instructions | None | None | None |
| Fine-tuning / RAG | None | None | None |
| Web browsing | Disabled | Disabled | Disabled |
| Memory / context | Disabled | Disabled | Disabled |

### **S2.3 Session management**

Each prompt was submitted as a single-turn query in a new, isolated conversation session. No conversation history, prior context, or follow-up messages were provided. Each of the 67 cases was submitted five times to each of the three models, yielding a total of 1,005 individual LLM queries (67 × 3 × 5). Between each submission, a new conversation session was initiated to prevent cross-contamination of context. The five runs per case were performed sequentially within the same session day for each model.

## **S3. Output Classification and Annotation**

### **S3.1 Classification procedure**

Each LLM response was classified into one of three categories: NOT TREAT, TREAT (COIL), or TREAT (CLIP). The prompt explicitly constrained outputs to this three-level format (see S1.2). In practice, all three LLMs adhered to the requested output format without exception; no responses required subjective interpretation or post-processing to extract the categorical recommendation. Classification was therefore deterministic: the recommendation and modality fields were extracted verbatim from each LLM response.

### **S3.2 Annotators**

Data extraction from MDT case notes was performed by two reviewers: MF and AN. Both reviewers independently extracted all 67 cases using a pre-specified extraction template. Discrepancies were resolved by consensus discussion; where consensus could not be reached, a third reviewer (FM) adjudicated.

### **S3.3 MDT reference standard extraction**

The MDT consensus decision for each case was extracted from the formal MDT meeting minutes recorded in the electronic medical record. MDT decisions were documented as a categorical outcome: NOT TREAT (observation), TREAT (COIL), or TREAT (CLIP). Where the MDT minutes recorded the decision unambiguously (e.g., “continue surveillance” or “proceed to coiling”), the classification was straightforward. No cases required subjective interpretation of the MDT decision.

## **S4. Compute Resources**

All LLM queries were performed via consumer web interfaces at no direct computational cost to the investigators. No local GPU, cloud compute, or API billing was involved. Approximate inference time per query ranged from 10 to 45 seconds depending on the model and thinking mode latency, with extended-thinking modes (Claude, Gemini) generally producing longer inference times.
