# Supplementary material for: Inter-Model Variability of ChatGPT, Gemini and Claude in Treatment Recommendations for Unruptured Intracranial Aneurysms
Source: Neurosurg Rev. 2026 Sep 16;49(1):569. doi: 10.1007/s10143-026-04498-1 (PMC13582248; doi:10.1007/s10143-026-04498-1)
Supplement: Supplementary file 2 [file 10143_2026_4498_MOESM2_ESM.docx]

# **TRIPOD+LLM Checklist — Completed Assessment**

| **Section** | **Item** | **Checklist Item** | **Status** | **Where Reported** |
| --- | --- | --- | --- | --- |
| **ABSTRACT** |  |  |  |  |
| **Title** | **2a** | Identify the study as evaluating the performance of an LLM, specifying the task, target population, and outcome. | **Reported** | Title. Title states "Inter-Model Variability of ChatGPT, Gemini and Claude in Treatment Recommendations for Unruptured Intracranial Aneurysms." Identifies the three LLMs, the inter-model evaluation task, the target population (UIA patients), and the outcome (treatment recommendation). |
| **Abstract** | **2b** | Provide a brief explanation of the healthcare context, use case and rationale for evaluating the LLM. | **Reported** | Abstract — Background. Explains that patients increasingly consult LLMs before specialist review and that whether frontier models agree with one another in nuanced clinical domains such as UIA management has not been characterised. |
| **Objectives** | **2c** | Specify study objectives, including whether the study describes LLM development, tuning, and/or evaluation. | **Reported** | Abstract — Background. Primary: quantify inter-model variability across ChatGPT, Gemini and Claude. Secondary: anchor each model's propensity against MDT consensus and UIATS. Framed as evaluation. |
|  | **2d** | Describe key elements of the study setting. | **Reported** | Abstract — Methods. Tertiary hospital, retrospective, January–December 2025, 67 patient cases. |
|  | **2e** | Detail all data used in the study, specify data splits and any selective use of data. | **Reported** | Abstract — Methods. 67 cases from neurovascular service. Baseline demographic, clinical, and aneurysm characteristics extracted into LLM prompts. No train/test split (evaluation study). |
|  | **2f** | Specify the name and version of LLM used. | **Reported** | Abstract — Methods. Claude Opus 4.6 (Anthropic), ChatGPT-5.4 (OpenAI), Gemini 3 Pro Thinking (Google DeepMind). |
|  | **2g** | Briefly summarize the LLM-building steps (fine-tuning, RLHF, etc.). | **N/A** | — Off-the-shelf commercial LLMs; no fine-tuning or custom training. |
|  | **2h** | Describe the specific tasks performed by the LLMs, highlighting key inputs and outputs. | **Reported** | Abstract — Methods. Standardised clinical vignettes submitted five times per case. Outputs: TREAT (COIL), TREAT (CLIP), or NOT TREAT with short reasoning. |
|  | **2i** | Specify evaluation datasets/populations, endpoint evaluated, and performance measures. | **Reported** | Abstract — Methods. 67 cases. Fleiss' κ (within-model reproducibility), pairwise Cohen's κ and McNemar's test (inter-model agreement and directional asymmetry), Cohen's κ (concordance with MDT/UIATS), McNemar's test (directional preference vs anchors). |
| **Results** | **2j** | Give an overall report and interpretation of main results. | **Reported** | Abstract — Results. Reports Fleiss' κ 0.837–0.860, pairwise inter-model κ 0.667–0.850 (ChatGPT–Gemini highest; Claude diverges from both), 19.4% non-unanimous, Claude sole outlier in 8/13 (conservative in 7), and directional propensities for each model. |
| **Discussion** | **2k** | Explicitly state any broader implications or concerns. | **Reported** | Abstract — Conclusion. Inter-model variability is asymmetric and directionally non-random; clinicians should anticipate AI-driven expectations. |
| **Other** | **2l** | Give registration number and name of registry (if relevant). | **N/A** | — Not a clinical trial. Ethics number and OSF registration (10.17605/OSF.IO/358MQ) reported in Statements and Declarations. |
| **INTRODUCTION** |  |  |  |  |
| **Background** | **3a** | Explain the healthcare context/use case and rationale, including references to existing approaches. | **Reported** | Introduction, paragraphs 1–2. UIA decision-making complexity, rise of patient-facing LLMs including ChatGPT Health, and gap in existing literature: single-model evaluations dominate; inherent treatment propensities of frontier LLMs and the clinical/aneurysmal factors driving them are uncharacterised. |
|  | **3b** | Describe the target population and intended use of the LLM in the care pathway, including intended users. | **Reported** | Introduction, paragraphs 1–2. Target population (adults with UIAs), care pathway (neurovascular MDT), context (patients consulting LLMs before specialist review) described. |
| **Objectives** | **4** | Specify study objectives, including whether the study describes development, fine-tuning, or evaluation. | **Reported** | Introduction, final paragraph. Primary: characterise inter-model variability (within- and between-model agreement). Secondary: anchor each model against MDT and UIATS to ascertain treatment tendencies and correlating patient/aneurysmal factors. Framed as evaluation. |
| **METHODS — DATA** |  |  |  |  |
| **Data** | **5a** | Describe the sources of data for evaluation datasets and rationale. | **Reported** | Materials and Methods — Case selection. Neurovascular service case records at Monash Health, January–December 2025. LLMs are commercial off-the-shelf models with proprietary training data. |
|  | **5b** | Describe relevant data points and provide quantitative/qualitative description of their distribution. | **Reported** | Materials and Methods — Data extraction; Results — Table 1. Table 1: comprehensive descriptive statistics across demographics, risk factors, functional status, aneurysm characteristics, and MDT/UIATS distributions. |
|  | **5c** | State the date of oldest and newest text used in development and evaluation datasets. | **Reported** | Materials and Methods — Case selection; Supplement S2.1. Evaluation: January–December 2025. LLM queries: March 2026. LLM training data cutoffs are proprietary. |
|  | **5d** | Describe data pre-processing and quality checking. | **Reported** | Materials and Methods — Data extraction; Supplement S3.2. De-identification described. Two reviewers (MF, AN) independently extracted all 67 cases; discrepancies resolved by consensus with FM as adjudicator. |
|  | **5e** | Describe how missing and imbalanced data were handled and reasons for omitting data. | **Reported** | Materials and Methods — Case selection. Exclusion criteria stated (ruptured, paediatric, insufficient documentation). Missing variables populated with "Not documented" rather than left blank (Supplement S1.3). |
| **METHODS — ANALYTICAL METHODS** |  |  |  |  |
| **Analytical Methods** | **6a** | Report the LLM name, version, and last date of training or use during inference. | **Reported** | Materials and Methods — Large language models and prompting strategy; Supplement S2.1. Claude Opus 4.6, ChatGPT-5.4, Gemini 3 Pro Thinking. Accessed via web interfaces. Prompts submitted March 2026. Extended thinking enabled for Claude and Gemini. |
|  | **6b** | Specify LLM architecture type, hyperparameters (temperature, length limits), prompt engineering, and inference settings. | **Reported** | Supplement S2.2 (Table). Full inference settings table: temperature (default 1.0, overridden by extended thinking for Claude), max tokens, seed, top-p/top-k all platform defaults and not user-configurable. Web browsing and memory disabled. No system instructions, fine-tuning, or RAG. |
|  | **6c** | Report LLM development process details (training, fine-tuning, alignment strategy). | **N/A** | — No custom development. Off-the-shelf models. |
|  | **6d** | Specify initial and post-processed output of the LLM. | **Reported** | Supplement S3.1. Outputs constrained to three-level format by prompt. All LLMs adhered without exception; classification was deterministic with recommendation and modality fields extracted verbatim. No post-processing. |
|  | **6e** | Provide details and rationale for classification and how thresholds were identified. | **Reported** | Materials and Methods — Outcomes; Supplement S3.1. Three-level classification (NOT TREAT, TREAT COIL, TREAT CLIP) defined by prompt constraints. Majority-vote across five runs. No probability thresholds. |
|  | **6f** | Include metrics capturing quality of generative outputs (consistency, relevance, accuracy). | **Reported** | Materials and Methods — Statistical analysis. Fleiss' κ (within-model reproducibility), pairwise Cohen's κ with bootstrap 95% CIs and McNemar's test (inter-model agreement and directional asymmetry), Cohen's κ and raw agreement (vs MDT/UIATS), McNemar's test (directional bias vs anchors). |
|  | **6g** | Report outcome metrics' relevance to downstream task and correlation to human evaluation. | **Reported** | Materials and Methods — Statistical analysis. Metrics measure inter-model agreement and agreement with real MDT consensus on actual patients. Landis and Koch framework cited. |
| **METHODS — LLM OUTPUT** |  |  |  |  |
| **LLM Output** | **7a** | Clearly define the outcome, how LLM predictions were calculated, and evaluation metrics. | **Reported** | Materials and Methods — Outcomes; Statistical analysis. Primary: pairwise inter-model agreement on binary and three-level treatment decision. Secondary: within-model reproducibility, agreement with MDT/UIATS, direction and magnitude of directional disagreement. |
|  | **7b** | If outcome assessment requires subjective interpretation, describe assessor qualifications and inter-assessor agreement. | **Reported** | Supplement S3.1–S3.3. All LLM outputs adhered to requested format; classification was deterministic, no subjective interpretation. Two reviewers (MF, AN) independently extracted all cases; consensus with FM adjudicator. MDT decisions extracted from formal minutes; no ambiguous cases. |
|  | **7c** | Specify how performance was compared to other LLMs, humans, and benchmarks. | **Reported** | Materials and Methods — Statistical analysis; Results — Tables 2 & 3; Fig. 2. Pairwise inter-LLM comparison (Cohen's κ with 95% CIs; McNemar for directional asymmetry); each LLM vs MDT and UIATS (Cohen's κ; McNemar); MDT vs UIATS as anchor-vs-anchor comparator. Table 2: LLM vs MDT and UIATS concordance. Table 3: pairwise inter-model agreement and directional asymmetry. Fig. 2: McNemar contingency tables. |
| **METHODS — ANNOTATION** |  |  |  |  |
| **Annotation** | **8a** | If annotation was done, report how text was labeled including annotation guidelines. | **Reported** | Supplement S3.1–S3.2. Data extraction using pre-specified template. LLM outputs classified deterministically via verbatim extraction. No annotation guidelines needed as no subjective interpretation required. |
|  | **8b** | Report how many annotators labeled the dataset(s) and proportion annotated by >1 annotator. | **Reported** | Materials and Methods — Data extraction; Supplement S3.2. Two reviewers (MF, AN) independently extracted all 67 cases (100% double-coded). Discrepancies resolved by consensus; FM available as adjudicator. |
|  | **8c** | Provide information on background/experience of annotators and inter-annotator agreement. | **Reported** | Supplement S3.2. Reviewers identified as MF and AN with institutional affiliations. |
| **METHODS — PROMPTING** |  |  |  |  |
| **Prompting** | **9a** | Provide details on processes used during prompt design, curation, and selection. | **Reported** | Supplement S1.1–S1.2; Fig. 1. Prompt developed de novo by AN and MF through iterative process. Three iterations of refinement. Piloted on non-study cases. Zero-shot structured-input approach. Full prompt reproduced verbatim in S1.2 and visualised in Fig. 1. |
|  | **9b** | Report what data were used to develop the prompts. | **Reported** | Supplement S1.1. Pilot cases selected from outside the January–December 2025 study period. Not included in the final 67-case analytic cohort. |
| **METHODS — OTHER** |  |  |  |  |
| **Summarization** | **10** | Describe preprocessing of data before summarization. | **N/A** | — Task is clinical decision classification, not summarization. |
| **Instruction Tuning** | **11** | If instruction tuning/alignment was used, describe instructions and interface. | **N/A** | — No instruction tuning. Off-the-shelf models via web interfaces. |
| **Compute** | **12** | Report compute or proxies (time, cost, machines, inference time, FLOPs). | **Reported** | Supplement S4. All queries via consumer web interfaces at no cost. Approximate inference time 10–45 seconds per query. Total 1,005 queries (67 × 3 × 5). |
| **Ethics** | **13** | Name IRB/ethics committee and describe participant consent or waiver. | **Reported** | Statements and Declarations — Ethics approval. Approved by institutional review board at Monash Health (RES-25-0000-885Q). Individual patient consent waived owing to retrospective, fully de-identified data. No identifiable information submitted to any LLM. |
| **OPEN SCIENCE** |  |  |  |  |
|  | **14a** | Give the source of funding and the role of the funders. | **Reported** | Statements and Declarations — Funding. "No funding was received for the conduct of this study or for the preparation of this manuscript." |
|  | **14b** | Declare conflicts of interest and financial disclosures. | **Reported** | Statements and Declarations — Competing interests. "The authors have no relevant financial or non-financial interests to disclose." |
|  | **14c** | Indicate where protocol can be accessed or state no protocol prepared. | **Reported** | Statements and Declarations — Registration. Study registered on OSF (10.17605/OSF.IO/358MQ). |
|  | **14d** | Provide registration information or state the study was not registered. | **Reported** | Statements and Declarations — Registration. OSF registration DOI: 10.17605/OSF.IO/358MQ. |
|  | **14e** | Provide details of the availability of study data. | **Reported** | Statements and Declarations — Data availability. "De-identified patient data and LLM output data are available from the corresponding author upon reasonable request, subject to institutional ethics approval." |
|  | **14f** | Provide details of the availability of code to reproduce results. | **Reported** | Statements and Declarations — Code availability. R scripts for statistical analysis available upon reasonable request. |
| **Public Involvement** | **15** | Provide details of patient/public involvement or state no involvement. | **N/A** | — Not required for evaluation studies. |
| **RESULTS** |  |  |  |  |
| **Participants** | **16a** | Describe the flow of patient data through the study. | **Reported** | Results, paragraph 1. Reports 67 cases with 99 aneurysms and MDT/UIATS outcome distributions. |
|  | **16b** | Report characteristics overall for each data source/setting, including key dates and sample size. | **Reported** | Results — Table 1. Comprehensive baseline characteristics at patient level (n = 67) and aneurysm level (n = 99). Dates (Jan–Dec 2025) and sample sizes stated. |
|  | **16c** | Show comparison of distribution of important predictors between development and evaluation data. | **N/A** | — No development/evaluation split. All cases used for evaluation. |
|  | **16d** | Specify number of participants and outcome events in each analysis. | **Reported** | Results, paragraphs 1–6. n = 67 for inter-model and LLM vs MDT analyses; n = 40 for LLM vs UIATS; n = 18 UNCLEAR subgroup; n = 13 non-unanimous; n = 10 universally discordant subgroup. |
| **Performance** | **17** | Report LLM performance according to pre-specified metrics and/or human evaluation. | **Reported** | Results — Tables 2, 3, 4; Fig. 2. Table 2: Fleiss' κ, Cohen's κ (binary/three-level) vs MDT and UIATS, raw agreement. Table 3: pairwise inter-model Cohen's κ with 95% CIs, McNemar inter-model directional asymmetry, three-way concordance. Fig. 2: McNemar contingency tables (LLM vs MDT, LLM vs UIATS, MDT vs UIATS). Table 4: 10 discordant case profiles. |
| **LLM Updating** | **18** | If applicable, report results from any LLM updating. | **N/A** | — No model updating performed. |
| **DISCUSSION** |  |  |  |  |
| **Interpretation** | **19a** | Give overall interpretation of results, including issues of fairness. | **Reported** | Discussion — Findings. Interprets asymmetric inter-model agreement (ChatGPT–Gemini near-identical; Claude divergent conservative outlier), and behavioural archetypes against MDT/UIATS (ChatGPT/Gemini pro-treatment, Claude conservative). Discusses sycophancy, constitutional AI, training data asymmetry, information asymmetry, and the doctor–patient–AI triad. Contextualises MDT conservatism within published UIA conservative-treatment rate range (38–70%). |
| **Limitations** | **19b** | Discuss limitations and effects on biases, statistical uncertainty, generalizability. | **Reported** | Discussion — Limitations. Single-centre; model snapshot and algorithmic drift; missing patient variables (comorbidity, anticoagulation, frailty, prior MDT decisions, adherence); rigid prompting; binary framing; no outcome validation. |
| **Usability** | **19c** | Describe challenges in using data for the task (representation, missingness, bias). | **Reported** | Discussion — Findings; Limitations. Information asymmetry between LLMs and MDT (Table 4). Training data bias. Missing comorbidity and emotional context. |
|  | **19d** | Define intended use for implementation (input, end-user, autonomy/oversight). | **Reported** | Discussion — Patient implications. Doctor–patient–AI triad discussed. Patients as end-users consulting LLMs before specialist review. Implications for clinical oversight and therapeutic relationship explored. |
|  | **19e** | Describe how poor quality or unavailable input data should be handled when implementing the LLM. | **Reported** | Discussion — Limitations and Future directions. Details regarding the lack of certain factors and poor input quality are discussed. |
|  | **19f** | Specify whether users will interact in handling input data, and what expertise is required. | **Reported** | Discussion — Patient implications. Patients (non-experts) consulting LLMs, sycophantic reinforcement, blinded evaluation preferences, AI-driven insistence and clinician dilemmas. |
|  | **19g** | Discuss next steps for future research. | **Reported** | Discussion — Future directions. Comorbidity integration (Charlson Comorbidity Index), personality-nuanced prompting, "be evidence-based" qualifiers, multicentre datasets to test stability of inter-model archetypes. |
